# Per and poly-fluoroalkyl substances and respiratory health in an Inuit community

**DOI:** 10.1186/s12940-024-01126-7

**Published:** 2024-10-12

**Authors:** Amira Aker, Yohann Courtemanche, Pierre Ayotte, Philippe Robert, Éric Gaudreau, Mélanie Lemire

**Affiliations:** 1 School of Public Health, Boston University, Boston, US; 2 Axe santé des populations et pratiques optimales en santé, Centre de recherche du CHU de Québec- Université Laval, Québec, QC Canada; 3 Département de médecine sociale et préventive, Université Laval, Québec, QC Canada; 4 Centre de Toxicologie du Québec, Institut National de Santé Publique du Québec, Quebec, Canada; 5 Institut de biologie intégrative et des systèmes (IBIS), Université Laval, Quebec, QC Canada; 6 Department of Epidemiology, Boston University School of Public Health, 715 Albany Street, Boston, MA 02118 US

## Abstract

**Background:**

Concentrations of plasma per and poly-fluoroalkyl substances (PFAS) are elevated in the Inuit population of Nunavik and may be causing adverse health effects. Respiratory health outcomes have been associated with PFAS, but have not been explored in Inuit communities. The aim of the study was to examine the association between PFAS and respiratory health outcomes, and the moderating role of nutritional biomarkers.

**Methods:**

We included up to 1298 participants of the Qanuilirpitaa? 2017 survey aged 16–80 years. Generalized regression models were used to estimate the associations between six individual PFAS congeners and four self-reported symptoms, four spirometry measures, and physician-diagnosed asthma. Outcomes associated with PFAS from single chemical models were further explored using Bayesian Kernel Machine Regression (BKMR). The modifying effect of n-3 PUFA in red blood cell quartiles and vitamin D deficiency were examined on the associations between PFAS and respiratory outcomes.

**Results:**

PFNA and PFOS were associated with asthma (odds ratio (OR) 1.61, 95% confidence interval (CI) 1.12, 2.32; OR 1.45 95% CI 1.04, 2.03). PFOA, PFNA, PFDA and PFHxS were associated with a decrease in the ratio between the forced expiratory volume in the first second and forced vital capacity (FEV1/FVC). No associations were observed with self-reported respiratory symptoms. No associations were observed between a PFAS mixture and asthma. Some associations were modified by nutritional factors, namely, stronger associations between PFOA and PFHxS and asthma with lower n-3 PUFA levels and stronger associations between PFDA, PFUnDA and PFOS and FEV1/FVC with vitamin D deficiency.

**Conclusion:**

These findings add to the growing literature on the impacts of PFAS on respiratory health, and the importance of their global regulation. Associations were modified by nutritional factors pointing to the nutritional value of traditional Inuit foods.

**Supplementary Information:**

The online version contains supplementary material available at 10.1186/s12940-024-01126-7.

## Background

PFAS (per and poly-fluoroalkyl substances) are a large group of synthetic chemicals used in a variety of consumer applications, industry processes, and fire-fighting foams for their ability to repel both water and oils [1]. They are environmentally persistent and can biomagnify in species, such that species higher up in the food chain tend to have higher PFAS concentrations [2, 3]. Due to their widespread use, there are several pathways of exposure, but consumption of aquatic foods contaminated with PFAS appears to be one of the most important sources of PFAS exposure in seafood-consuming populations [3–5].

The Inuit population of Nunavik, Quebec has among the highest overall PFAS exposures in Canada and across other Arctic populations [4, 6]. Inuit traditional foods are harvested and hunted from the land, river and seas, and species traditionally consumed include beluga whale, seal, caribou and various types of fish, mollusks and wild berries. The consumption of some of these highly nutritious and culturally essential traditional foods, including beluga and seal, has been associated with elevated PFAS biomarker concentrations in Nunavik [7]. Additionally, the increased PFAS concentrations are largely driven by high concentrations of long-chain perfluorocarboxylic acids (PFCAs) with nine or more carbons (C ≥ 9), which tend to be more environmentally persistent and present greater bioaccumulation and biomagnification potential in wildlife [8]. For example, perfluorononanoic acid (PFNA) plasma levels were up to seven-fold higher in Nunavimmiut (Inuit living in Nunavik) compared to the general Canadian population [9].

PFAS have been under additional scrutiny over the last few years due to their documented effects on human health, including impacts on cardiometabolic, developmental, endocrine, and immunological health [3, 10]. However, the impacts of PFAS (particularly long-chain PFCAs) on Nunavimmiut health are still unclear.

Respiratory health is a major public health concern in Nunavik. A recent representative survey of Nunavimmiut aged 16 years and over, Qanuilirpitaa? 2017 Nunavik Health Survey (Q2017), documented a high prevalence of chronic cough, wheezing, and hospitalization and mortality rates from respiratory causes [11]. For example, the prevalence of chronic cough in Nunavik was twofold the prevalence among Canadians in the longitudinal Canadian Cohort Obstructive Lung Disease cohort. Although asthma rates in Nunavik were lower than those reported in the general Canadian population, the higher prevalence of adverse respiratory health outcomes and obstructive pattern on spirometry indicate an underdiagnosis of asthma in Nunavik [11].

PFAS distribute and accumulate in the lungs [12, 13], and have been associated with respiratory outcomes, including asthma and allergies, in several populations [14–17]. Although results pertaining to PFAS and asthma have been mixed and inconclusive [10, 18], mechanistic studies suggest that PFAS cause inflammation in lung cells and tissues [19–21], suppress neutrophil respiratory bursts [22], and may activate innate immune systems [23], thus providing biological plausibility to an association between PFAS and adverse respiratory outcomes.

Several factors were associated with the increased respiratory symptoms in Nunavik, including smoking (72% smoking prevalence [24]), obesity, housing conditions, and socioeconomic status [11]. Vitamin D, omega-3 fatty acids, and fruits/vegetable consumption may be protective of respiratory health [25, 26], and seafood and marine mammal consumption are major sources of vitamin D and omega-3 fatty acids in Nunavik [27, 28]. Surprisingly, however, increased consumption of marine mammals and fish was associated with an increase in respiratory symptoms in Nunavik [11]. This may be due to the strong association between environmental contaminants and these foods, including PFAS and mercury [27, 29]. The interplay between environmental contaminants and nutrients from similar sources is important to consider in the relationship between PFAS and respiratory health.

The objective of this study was to examine the associations between PFAS congeners and adverse respiratory symptoms, airway obstruction, and asthma in Nunavimmiut using who participated in the Q2017 survey. Due to the nutritional value of PFAS exposure sources in Nunavik, we also explored the influence of dietary factors.

## Methods

### Study Population

The Qanuilirpitaa? 2017 Nunavik Health Survey (Q2017) survey was conducted from August 19 to October 5, 2017, and covered all 14 Nunavik villages situated across three ecological regions. Accessing these isolated coastal villages was made possible through the Canadian Coast Guard Icebreaker, Amundsen, which facilitated the collection of data as participants were invited aboard the ship. The survey aimed to include all Nunavik permanent residents aged 16 years and above, and a structured stratified proportional model was employed for selecting respondents [30]. A total of 1326 residents were successfully recruited for the study. Data collection encompassed comprehensive questionnaires, clinical measurements, as well as the collection of biological samples such as urine and blood.

The Q2017 survey adhered to the principles of OCAP^®^ (Ownership, Control, Access, and Possession), ensuring respectful collaboration with various Nunavik organizations throughout the entire process. Inuit colleagues and partners and the Q2017 Nunavik Surveys Committee contributed to all discussions and result interpretation. The Q2017 Steering Committee, comprising members from diverse Nunavik organizations and the 14 municipalities, oversaw all research utilizing the dataset. Additionally, several co-authors played roles in co-designing, managing, and implementing the data collection process for Q2017. The Q2017 survey received ethical approval from the Comité d’éthique de la recherche du Centre Hospitalier Universitaire de Québec - Université Laval (#2016–2499 and #2016–2499-21).

## Exposure biomarkers

The blood samples were collected by research nurses via venipuncture using K2-EDTA vacutainers (Fisher Scientific). Subsequently, the collected samples were centrifuged at 2000×g for 10 min, and the resulting plasma was transferred into polypropylene tubes for storage at − 20 °C until further analysis.

The analysis of perfluoroalkyl and polyfluoroalkyl substances (PFAS), including perfluorobutanoic acid (PFBA), perfluorohexanoic acid (PFHxA), perfluorooctanoic acid (PFOA), perfluorononanoic acid (PFNA), perfluorodecanoic acid (PFDA), perfluoroundecanoic acid (PFUnDA), perfluorobutane sulfonic acid (PFBS), perfluorohexane sulfonic acid (PFHxS), and perfluorooctane sulfonic acid (PFOS), was conducted at the Centre de Toxicologie du Québec (CTQ) of the Institut national de santé publique du Québec (INSPQ), which holds accreditation from the Canadian Association for Environmental Analytical Laboratories and ISO 17,025.

For the PFAS analysis, plasma samples (100 µL) were enriched with labeled internal standards (PFBA-^13^C4, PFHxA-^13^C6, PFOA-^13^C4, PFNA-^13^C9, PFDA-^13^C9, PFUndA-^13^C7, PFHxS-^13^C3, and PFOS-^13^C4) and acidified using a 50% formic acid solution. Subsequently, solid phase extraction (SPE) was employed with SiliaPrepX WAX cartridges 100 mg/3 mL (Silicycle; Québec, Canada) to extract the analytes. The cartridges were initially washed with 5% NH_4_OH in methanol to remove contaminants and then conditioned with methanol and 2% formic acid before processing the samples. Following the loading of samples on the cartridges, a washing step with a 2% formic acid solution and methanol was performed, and the analytes were subsequently eluted using 3 mL of 5% NH_4_OH in methanol. The extracts were evaporated to dryness and then reconstituted in 1 mL of 5 mM ammonium acetate in 20% acetonitrile.

Analysis of the samples was carried out using Ultra Performance Liquid Chromatography (UPLC Waters Acquity) coupled with tandem mass spectrometry (MS/MS Waters Xevo TQ-S) (Waters, Milford, MA, USA) operating in the multiple reaction monitoring (MRM) mode with an electrospray ion source in negative mode. The analytical column utilized was an ACE EXCEL C18-PFP 100 mm × 2.1 mm, 2.0 μm (ACE; Aberdeen, Scotland). The mobile phase consisted of an acetonitrile: H_2_O gradient (10:90) with 5 mM ammonium acetate, which was gradually changed to 100% acetonitrile with 5 mM ammonium acetate over a 7.0-minute run at a flow rate of 0.5 mL/min. This analytical method represents an improved version of the one previously described by Caron-Beaudoin et al. (2020), and both methods were cross-validated, demonstrating equivalent results. The limit of detection (LOD) for the perfluorinated compounds ranged between 0.01 and 0.07 µg/L. Intra-day precision varied between 2.0 and 3.8%, while inter-day precision varied between 3.8 and 5.0%, depending on the analytes. To monitor contamination, two laboratory blanks containing demineralized water were included in each analytic sequence. Polypropylene materials, such as tubes and microvials, were utilized to prevent the adsorption of PFAAs, and most of the other materials (glassware, pipette tips, etc.) were washed with methanol before use to minimize contamination. Additionally, an isolator column was inserted before the analytical column to control potential contaminants originating from the UPLC system.

To ensure the quality of the analyses, internal reference materials, such as the certified reference material SRM-1958 from the National Institute of Standards and Technology (NIST; Gaithersburg, MD), and in-house quality controls (QCs) for PFAS were employed. The overall quality and accuracy of the analytical method were monitored through participation in interlaboratory programs, including the AMAP External Quality Assessment Scheme (Centre de Toxicologie du Québec (CTQ), Institut National de Santé Publique du Québec (INSPQ), Québec, Canada) for the analytes PFHxA, PFOA, PFNA, PFDA, PFUnDA, PFHxS, and PFOS, as well as the German External Quality Assessment Scheme (G-EQUAS; Erlangen, Germany) for the analytes PFOA and PFOS.

Furthermore, the analysis of red blood cell fatty acid composition was conducted at the Laboratory of Nutritional Lipidomics of the University of Waterloo, Ontario, employing a Varian 3900 gas chromatograph equipped with a 15 m DB-FFAP capillary column (df = 0.10 μm) and a flame-ionisation detector. Concentrations of RBC eicosapentaenoic acid, docosahexaenoic acid, and docosapentaenoic acid were summed and expressed as a percentage of total fatty acids. This variable was further categorized into quartiles (n-3 PUFA). Serum vitamin D were determined using a MODULAR ANALYTICS e170 from Roche Diagnostics GmbH (Mannheim, Germany).

Whole blood total mercury (a surrogate for methylmercury exposure in fish and marine mammal eating populations) concentrations were measured using inductively coupled plasma mass spectrometry (ICP-MS) with a NexION^®^ instrument from PerkinElmer (Waltham, MA, USA) and were analyzed at the Centre de toxicologie du Québec (Quebec, QC). Further details have been described elsewhere [31].

Organochlorines, including aldrin, α-chlordane, γ-chlordane, cis-nonachlor, trans-nonachlor, oxychlordane, p,p’-dichlorodiphenyldichloroethylene (p, p’-DDE), p,p’-dichlorodiphenyltrichloroethane (p, p’-DDT), β-hexachlorocyclohexane (HCH), hexachlorobenzene, mirex, toxaphene no. 26, toxaphene no. 50 and 24 congeners of polychlorinated biphenyls (PCB IUPAC #: 28, 52, 66, 74, 99, 101, 105, 118, 128, 138, 146, 153, 156, 163, 167, 170, 178, 180, 183, 187, 194, 201, 203 and 206) and polybrominated compounds (PBDE IUPAC #: 15, 17, 25, 28, 33, 47, 99, 100, 153; polybrominated biphenyl IUPAC # 153), were quantified in plasma samples using the CTQ’s method E-446, described in Fisher et al. (2016). Briefly, compounds were extracted using a liquid-liquid extraction with a mixture of ammonium sulfate: denaturated alcohol: hexane (1:1:3). Thereafter, the extracts were concentrated, purified on Florisil and then analyzed by gas chromatography/mass spectrometry (Agilent Technologies) after a negative chemical ionization.

## Outcome measurement

Respiratory health was measured via questionnaires (self-reported symptoms), spirometry measures, and medical records.

Self-reported symptoms included wheezing, chronic cough, chronic sputum, and breathlessness. Wheezing was defined as “wheezing or whistling in the chest at any time during the last 12 months”, and is considered an indicator of asthma [32]. There is no direct translation of the term “wheezing” in Inuktitut, as such this was translated to “laboured breathing” () or “whistling sound” (). The reliability of this indicator was previously tested against airway obstruction and reported an OR 2.26 (95% CI 1.46, 3.48) [11]. The most commonly used epidemiological definition for chronic cough was adopted, defined as a “cough on most days for three months each year” [33]. Chronic sputum was defined as “bringing up mucus on most days for three months each year, if the person usually brings up mucus from his chest when she does not have a cold”, and breathlessness was defined as “walking slower than people of the same age on the level because of breathlessness or having to stop for breath when walking at its own pace on the level”, corresponding to the second stage of the Modified Medical Research Council scale [34].

The main spirometry measures included in this analysis were the forced expiratory volume in the first second (FEV1) and forced vital capacity (FVC). The FEV1/FVC ratio below the lower limit of normal (LLN) (a continuous variable) was used to examine airway obstruction [35]. No reference equation exists for the Inuit population, however the FEV1/FVC ratio shows minimal variation across ethnic groups [36]. The LLN was calculated based on age and sex using the NHANES III reference equation for Caucasians [11, 37]. A second airway obstruction variable was calculated based on the fixed ratio recommended by GOLD (< 0.7) (a binary variable). It serves as an epidemiological definition for Chronic Obstructive Pulmonary Disease (COPD) in adults over 35 years old and can also be indicative of asthma, suboptimal lung development, or early lung decline [38].

Medical files (outpatient visits, emergency visits and hospitalizations) were reviewed by trained research nurses for physician diagnoses of asthma, COPD (including emphysema and chronic bronchitis), tuberculosis (latent or active/pulmonary) and hospitalization for respiratory infection during childhood (before the age of 5 years). Diagnosis of asthma or COPD in medical file are not necessarily supported by spirometry because it is not widely available in Nunavik.

## Covariates

Participant sociodemographic characteristics were gathered through questionnaires. The covariates of interest included age (years), education level (< 9th Grade level, at least some high school, at least some college), personal income (<$20,000, $20,000-$59,999, >$60,000), marital status (married or common law, single), smoking status (never, former, current smoker < 15 pack-years, current smoker ≥ 15 pack-years), second-hand smoking exposure (< 1/month, 1/week – 1/month, nearly everyday), marijuana use (rarely, 1–3/month, ≥ 1/week), food security (secure, moderately insecure, severely insecure), house crowding (≤ 1 person-per-room, > 1 person-per-room), vitamin D concentrations, n-3 PUFA in red blood cell quartiles (a marker of seafood and marine mammal consumption), fruit and vegetable consumption (< 5 times/day, ≥5times/day) [39], waist circumference quartiles, and blood total mercury concentrations (Supplementary Fig. 1).

Dietary factors associated with respiratory outcomes and PFAS were included in the models. Among the main exposure sources of vitamin D and n-3 PUFA in red blood cells are marine mammals and seafood [27, 28, 40, 41] which are also the main exposure sources of PFAS.

Further details on these covariates (calculations and questionnaire responses) have been described elsewhere [11, 29]. In place of body mass index (BMI), we used waist circumference as a proxy for body size. This decision was influenced by the fact that BMI tends to overestimate the prevalence of obesity in Inuit populations [42]. Some recent evidence suggests a link between exposure to mercury and respiratory health [43–45]. Given that elevated mercury exposure is common in Nunavik due to the high mercury concentrations found in some marine mammals and fish consumed by Inuit [27, 31], we included whole blood total mercury levels as a potential confounding factor.

### Statistical analysis

Multiple imputation using fully conditional specification was utilized to impute missing data with plausible values. The technique involved multivariate linear regression for imputing continuous variables, logistic regression for imputing binary variables and multinomial logistic regression for imputing categorical variables [46]. Predictions for each variable were made with the inclusion of all other variables used in the study, including socioeconomic, lifestyle and respiratory predictors. Given that 57% of participants had missing values for at least one variable (typically a few variables), a total of 60 complete datasets were imputed. Each imputed dataset underwent separate analysis, and the results were subsequently pooled using the “mianalyze” SAS procedure. Descriptive data from non-imputed and imputed datasets showed high similarity. Notably, respiratory outcomes were not imputed and participants with missing outcome data (*n* = 38 for wheezing, *n* = 39 for chronic cough, *n* = 208 for airway obstruction) were excluded from the analysis.

Plasma levels of all PFAS congeners were log2-transformed. Geometric means and their corresponding 95% confidence intervals (CIs) were calculated for exposure variables, and proportions were calculated for the categorical covariate and outcome variables. The percentage of samples with detectable PFBA, PFHxA, and PFBS concentrations were low with > 80% of values below the LOD and were not included in further analyses. Study demographics were explored in the non-imputed dataset to compare the distributions in imputed and non-imputed datasets. We also calculated the distributions in an imputed and weighted dataset. Survey weights were included to account for the complex stratified sampling strategy and the non-response rate [9, 47].

Binomial generalized linear models was conducted to explore associations between individual PFAS congeners and respiratory outcomes wheezing, airway obstruction, and asthma. The study focused on wheezing, chronic cough, airway obstruction, and asthma because these were considered to be respiratory symptoms of concern in the Nunavik context [11]. However, we also examined associations between PFAS and chronic sputum, breathlessness, and having any respiratory symptom in supplementary analyses. Beta coefficients were exponentiated to calculate the odds ratio (and associated 95% CI) for a doubling of the exposure. Additionally, multivariate linear regression models were used to examine the associations between PFAS congeners and FEV1, FVC and the FEV1/FVC ratio. Beta coefficients were exponentiated to calculate the unit outcome change for every doubling of the exposure.

Covariates were included in two stages. The first adjusted model controlled for sociodemographic and some lifestyle variables: sex, age, personal income, marital status, smoking status, second-hand smoking, marijuana use, waist circumference, overcrowding, and food security. The second adjusted models were further adjusted for n-3 PUFA red blood cell quartiles, vitamin D concentrations, fruit/vegetable intake, and blood mercury concentrations to account for other nutrient and environmental contaminants detected in PFAS exposure sources. No issues of multicollinearity emerged after checking correlation matrices and model variance inflation factors (VIF) values.

We also examined the modifying effect of nutrition on the associations between PFAS and respiratory outcomes, namely n-3 PUFA in red blood cell quartiles and vitamin D deficiency (defined as vitamin D levels < 30 ng/mL) (described above). An interaction term was included in all models between individual PFAS congeners and each of the nutritional variables. If a significant interaction was observed (p-value < 0.05), we stratified the models by the nutritional categories.

Outcomes associated with PFAS from single chemical models were further explored using mixture analyses. The association between PFAS congeners and respiratory outcomes was modeled by Bayesian Kernel Machine Regression (BKMR), which allows for correlation, non-linearity and interactions between exposures. The BKMR model relies on a function of PFAS congeners to estimate the exposure-response surface used to compute contrasts of interest and inferences. For example, the overall effect of the exposure mixture can be obtained by comparing the point estimates of the exposure-response surface when all mixture components are set to a particular quantile versus all set at a reference level (50th quantile). Furthermore, exposure-response functions can be obtained to visualize non-linear relationships and to investigate interactions between mixture components by estimating the bivariate exposure-response function by varying level (25th, 50th and 75th quantiles) of another component. The bkmr R package [48] was used to estimate the covariate-adjusted exposure-response surface and to calculate credible intervals (CI) for inferences. Missing data were imputed in 60 datasets and a separate BKMR model with 10,000 iterations in the Markov chain Monte Carlo algorithm was fit for each dataset. The first 5,000 iterations were discarded and 20% of remaining iterations were used to compute posterior means and variances. Estimates of each dataset were combined using Rubin’s method [49]. The theoretical background of the BKMR method and implementation with this package has been described in detail elsewhere [48, 50].

### Sensitivity analyses

According to the directed acyclic diagram (DAG) created (Supplementary Fig. 1), adjusting for participants with recent respiratory infections or surgery may lead to bias by collider. We did, however, conduct a sensitivity analysis excluding participants with emphysema or recent/past history of tuberculosis. Furthermore, asthma is sometimes misdiagnosed with chronic obstructive pulmonary disease (COPD) in older adults. As such, we conducted another analysis expanding the asthma definition to include COPD diagnosis, self-reported asthma, self-reported asthmatic bronchitis, or self-reported allergic bronchitis. We also replaced the current smoking status variable with cotinine levels and found no differences in the results. Other persistent organic pollutants (POPs) are detected in high concentrations in Nunavik, and we conducted another sensitivity analysis that included each of these POPs in the model individually to test for potential confounding. The POPs considered included chlordane, DDE, βHCH, hexachlorobenzene, mirex, sum of toxaphene no. 26 and toxaphene no. 50, and the sum of 24 congeners of polychlorinated biphenyls. The cross-sectional nature of the study limits our ability to establish temporality, particularly with regards to asthma. While asthma diagnosis in adulthood is common [51], most people with asthma are diagnosed as children [52]. To better ascertain temporality, we conducted additional sensitivity analyses by restricting the analysis of PFAS and asthma to those aged 16–20 years. Given the persistent nature of PFAS, we assumed that PFAS levels remained relatively stable throughout childhood. This assumption is strengthened in the Nunavik context since the main exposure source of PFAS is consumption of country (traditional) foods and is reflected by ties to culture, so we assumed that the consumption of country versus market foods remained relatively stable in childhood as well.

All regression model analyses were performed using SAS 9.4 (SAS Institute, Cary, NC, USA) and BKMR models were performed using R 4.3.1 software.

## Results

The study population (*N* = 1326) had a mean age of 37.5 years and 65.8% were female versus 34.2% males (Table 1). After imputation, only 15.4% of the population had an income of >$60,000 and a corresponding 14.7% finished at least some college. Approximately 78% of the study population were current smokers, and 21.5% smoked ≥ 15 pack-years. An additional 48.4% were exposed to second-hand smoke everyday and almost half reported smoking marijuana ≥ 1/week. Only 33.8% of the study population reported being food secure. Over a quarter of participants reported wheezing, and approximately a fifth reported chronic cough, chronic sputum, or breathlessness (Table 2). PFOS and PFNA had the highest concentrations compared to other PFAS compounds (Table 3).

**Table 1:** Study population descriptives in the imputed dataset from Q2017, Nunavik, Quebec (*N* = 1326)

Characteristic	Total Population
**Age (years (SD))**	37.5 (16.4)
**Income**	
<$20,000	53.7
$20,000-$59,999	31.0
>$60,000	15.4
**Education**	
< Grade 9	38.6
At least some high school	46.7
At least some college	14.7
**Marital status**	
Married or living with partner	49.1
Single	50.9
**Smoking**	
Never	10.6
Former	11.4
Current < 15 pack-years	56.5
Current ≥ 15 pack-years	21.5
**Second hand smoke**	
< 1/month	66.4
1/week – 1/month	7.1
Nearly everyday	26.5
**Marijuana use**	
Rarely/Never	30.0
1–3/month	21.6
≥ 1/week	48.4
**Food security**	
Food secure	33.8
Moderately insecure	47.2
Severely insecure	19.0
**Housing crowding)**	
≤ 1 person-per-room	67.3
> 1 person-per-room	32.7
**Fruit/vegetable consumption**	
< 5 times/day	83.2
≥ 5 times/day	16.8
**n-3 PUFA in red blood cells (Mean (SD)) (µg fatty acid/100 mg erythrocytes)**	7.7 (2.5)
Q1 (< 5.83)	25.0
Q2 (5.83–7.40)	25.0
Q3 (7.40–9.39)	25.0
Q4 (> 9.39)	25.0
**Vitamin D levels (Mean nmol/L (SD))**	66.4 (1.2)
**Waist Circumference** ^**a**^ **(Mean (SD)) (cm)**	93.3 (16.3)
Q1	24.6
Q2	25.0
Q3	25.2
Q4	25.3

^a^ Waist circumference quartiles based on sex. Females : Q1 < 82, 82 ≤ Q2 < 94, 94 ≤ Q3 < 105, Q4 ≥ 105; Males : Q1 < 79, 79.5 ≤ Q2 < 86.75, 86.75 ≤ Q3 < 103.5, Q4 ≥ 103.5 cm

^b^ Calculated using the LLN NHANES equation

**Table 2:** Distribution of respiratory outcomes in the imputed dataset from Q2017, Nunavik, Quebec (*N* = 1326)

Outcome	Total Population	16–20 years	21–35 years	≥ 36 years
	Percentage of population	Percentage of population	Percentage of population	Percentage of population
**Wheeze**	27.2	22.0	27.6	29.1
Missing	2.1	4.5	0.7	2.0
**Chronic cough**	20.7	16.1	15.1	25.1
Missing	2.1	4.5	0.7	2.0
**Chronic sputum**	21.3	21.7	19.3	21.4
Missing	2.1	4.5	0.7	2.0
**Breathlessness**	20.7	18.0	18.5	22.3
Missing	2.1	4.5	0.7	2.0
**Any respiratory symptom**	40.9	38.2	35.1	38.5
Missing	8.6	7.5	6.1	10.6
**Asthma**	3.7	5.6	3.9	2.8
Missing	6.6	3.4	5.4	8.6
**Airway obstruction**	16.0	15.0	14.2	12.2
Missing	16.3	16.9	14.9	17.0
	Mean of the population	Mean of the population	Mean of the population	Mean of the population
**FEV1** ^**a**^ **(L)**	2.50	2.94	2.73	2.17
Missing (%)	16.3	16.9	14.9	17.0
**FVC** ^**b**^ **(L)**	3.04	3.37	3.25	2.78
Missing (%)	16.3	16.9	14.9	17.0
**FEV1/FVC** ^**c**^ **(%)**	72.31	76.38	74.44	69.25
Missing (%)	16.3	16.9	14.9	17.0

a FEV1: the forced expiratory volume in the first second

b FVC: forced vital capacity

c FEV1/FVC ratio below the lower limit of normal (LLN)

**Table 3:** Distribution of exposure biomarkers in the Q2017 survey, Nunavik, Quebec (*N* = 1326). All units in µg/L

	GM (95% CI)	25th percentile	75th percentile
**PFBA**	< LOD	< LOD	< LOD
**PFHxA**	< LOD	< LOD	< LOD
**PFOA**	0.98 (0.98, 0.99)	0.68	1.4
**PFNA**	3.81 (3.79, 3.82)	2.4	6.0
**PFDA**	0.70 (0.69, 0.70)	0.4	1.2
**PFUnDA**	0.72 (0.72, 0.72)	0.43	1.3
**PFBS**	< LOD	< LOD	< LOD
**PFHxS**	0.59 (0.59, 0.60)	0.34	0.97
**PFOS**	4.94 (4.91, 4.97)	2.7	8.8
**Mercury**	8.75 (8.69, 8.82)	24.0	90.0

PFAS were not associated with wheezing, chronic cough or airway obstruction (fixed ratio recommended by GOLD) even after adjustment for dietary factors and blood mercury (Table 4). Similarly, PFAS were not associated with chronic sputum, breathlessness or having any respiratory symptom (Supplementary Table 2). PFNA and PFOS were associated with increased odds of asthma after adjustment for sociodemographic variables, dietary factors, and blood mercury (OR 1.61 95% CI 1.12, 2.32; OR 1.45 95% CI 1.04, 2.03). PFOA had the largest strength of association, albeit with marginal significance (OR 1.65 95% CI 0.96, 2.83). PFDA, PFUnDA, and PFHxS were also associated with increased odds of asthma, but none reached statistical significance. PFOA, PFNA, PFDA, and PFHxS were associated with decreases in the FEV1/FVC ratio, although the strength of the associations decreased after adjustment for covariates (Table 5). There was no evidence of interaction by sex (data not shown).

**Table 4:** Odds ratios and their respective 95% confidence intervals of wheezing, chronic cough, asthma and airway obstruction for every doubling of individual PFAS congener from the Q2017 survey in Nunavik, Quebec

	Unadjusted	Adjusted I	Adjusted II
**Wheezing**			
PFOA	1.07 (0.92, 1.25)	0.97 (0.79, 1.18)	1.01 (0.80, 1.27)
PFNA	0.94 (0.83, 1.07)	0.91 (0.79, 1.04)	0.91 (0.77, 1.09)
PFDA	1.00 (0.90, 1.11)	0.97 (0.86, 1.10)	1.00 (0.82, 1.22)
PFUnDA	0.97 (0.88, 1.08)	0.95 (0.84, 1.07)	0.95 (0.77, 1.16)
PFHxS	1.03 (0.93, 1.15)	0.90 (0.77, 1.04)	0.89 (0.74, 1.07)
PFOS	0.99 (0.90, 1.1)	0.94 (0.83, 1.06)	0.93 (0.78, 1.10)
**Chronic cough**			
PFOA	1.21 (1.03, 1.42)	1.11 (0.88, 1.40)	1.09 (0.84, 1.41)
PFNA	1.11 (0.96, 1.28)	1.03 (0.87, 1.21)	0.97 (0.79, 1.18)
PFDA	1.14 (1.02, 1.28)	1.06 (0.93, 1.22)	0.93 (0.75, 1.16)
PFUnDA	1.15 (1.02, 1.29)	1.08 (0.94, 1.23)	0.95 (0.76, 1.19)
PFHxS	1.17 (1.04, 1.31)	1.06 (0.90, 1.25)	0.99 (0.81, 1.21)
PFOS	1.10 (0.99, 1.23)	1.05 (0.92, 1.20)	0.95 (0.79, 1.14)
**Asthma**			
PFOA	1.17 (0.87, 1.58)	1.61 (0.97, 2.67)	1.65 (0.96, 2.83)
PFNA	1.24 (0.97, 1.57)	**1.46 (1.08**,** 1.98)**	**1.61 (1.12**,** 2.32)**
PFDA	0.94 (0.76, 1.17)	1.12 (0.84, 1.49)	1.37 (0.90, 2.07)
PFUnDA	0.92 (0.74, 1.13)	1.06 (0.81, 1.38)	1.27 (0.83, 1.95)
PFHxS	0.93 (0.77, 1.12)	1.13 (0.82, 1.58)	1.24 (0.84, 1.83)
PFOS	0.99 (0.81, 1.20)	1.18 (0.91, 1.54)	**1.45 (1.04**,** 2.03)**
**Airway Obstruction**			
PFOA	1.06 (0.87, 1.29)	0.90 (0.69, 1.18)	0.83 (0.61, 1.12)
PFNA	0.93 (0.79, 1.10)	0.96 (0.80, 1.15)	0.85 (0.68, 1.07)
PFDA	0.98 (0.86, 1.12)	1.03 (0.88, 1.20)	0.82 (0.63, 1.06)
PFUnDA	1.03 (0.90, 1.17)	1.07 (0.92, 1.25)	0.88 (0.68, 1.15)
PFHxS	1.05 (0.91, 1.22)	0.96 (0.78, 1.18)	0.83 (0.65, 1.07)
PFOS	0.98 (0.86, 1.12)	1.01 (0.86, 1.18)	0.85 (0.68, 1.06)

Number of individuals in models: wheezing and chronic cough *N* = 1298; asthma *N* = 1239; airway obstruction *N* = 1110.

Adjusted I models are adjusted for sex, age, personal income, marital status, smoking status, second-hand smoking, marijuana use, waist circumference, overcrowding, and food security.

Adjusted II models are further adjusted for n-3 PUFA quartiles, vitamin D, fruit/vegetable intake, and mercury.

Airway obstruction is defined by the fixed ratio recommended by GOLD (0.7).

**Table 5:** Relative risk and respective 95% confidence intervals of FEV1, FVC, and FEV1/FVC ratio below the lower limit of normal (LLN) for every doubling of individual PFAS congener and from the Q2017 survey in Nunavik, Quebec

	Unadjusted	Adjusted I	Adjusted II
**FEV1**			
PFOA	**-0.08 (-0.12**,** -0.04)**	0.02 (-0.02, 0.05)	**0.05 (0.01**,** 0.08)**
PFNA	**-0.15 (-0.18**,** -0.12)**	-0.001 (-0.03, 0.02)	0.005 (-0.02, 0.03)
PFDA	**-0.19 (-0.22**,** -0.17)**	**-0.02 (-0.03**,** -0.004)**	-0.02 (-0.05, 0.02)
PFUnDA	**-0.19 (-0.21**,** -0.16)**	-0.02 (-0.04, 0.005)	-0.01 (-0.05, 0.02)
PFHxS	**-0.12 (-0.15**,** -0.09)**	-0.02 (-0.04, -0.01)	0.02 (-0.01, 0.05)
PFOS	**-0.15 (-0.18**,** -0.12)**	-0.01 (-0.02, -0.002)	0.005 (-0.03, 0.03)
**FVC**			
PFOA	**0.04 (-0.01**,** 0.08)**	**-0.03 (-0.05**,** -0.01)**	0.01 (-0.01, 0.04)
PFNA	**-0.13 (-0.17**,** -0.09)**	**-0.03 (-0.04**,** -0.01)**	-0.01 (-0.03, 0.01)
PFDA	**-0.18 (-0.21**,** -0.15)**	**-0.01 (-0.01**,** -0.0002)**	-0.003 (-0.03, 0.02)
PFUnDA	**-0.18 (-0.21**,** -0.15)**	**-0.03 (-0.05**,** -0.01)**	0.003 (-0.02, 0.03)
PFHxS	-0.03 (-0.07, 0.004)	**-0.02 (-0.04**,** -0.01)**	0.01 (-0.01, 0.03)
PFOS	**-0.11 (-0.14**,** -0.08)**	-0.01 (-0.01, 0.001)	0.01 (-0.01, 0.03)
**FEV1/FVC**			
PFOA	**-2.55 (-2.77**,** -2.33)**	-0.01 (-0.03, 0.019)	**-0.02 (-0.03**,** -0.004)**
PFNA	**-1.17 (-1.38**,** -0.96)**	-0.01 (-0.03, 0.006)	**-0.01 (-0.02**,** -0.002)**
PFDA	**-1.19 (-1.36**,** -1.02)**	**-0.01 (-0.02**,** -0.0008)**	**-0.01 (-0.02**,** -0.0003)**
PFUnDA	**-0.94 (-1.11**,** -0.76)**	-0.02 (-0.04, 0.003)	-0.01 (-0.02, 0.004)
PFHxS	**-2.09 (-2.23**,** -1.95)**	**-0.02 (-0.03**,** -0.002)**	**-0.01 (-0.02**,** -0.0004)**
PFOS	**-1.41 (-1.57**,** -1.26)**	-0.01 (-0.01, 0.002)	-0.01 (-0.02, 0.003)

Number of individuals in models = 1110

Adjusted I models are adjusted for sex, age, personal income, marital status, smoking status, second-hand smoking, marijuana use, waist circumference, overcrowding, and food security.

Adjusted II models are further adjusted for n-3 PUFA quartiles, vitamin D, fruit/vegetable intake, and mercury.

**Fig. 1:**
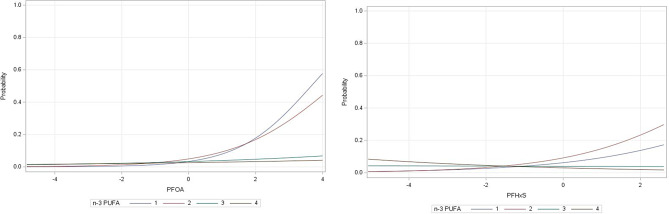
Association between PFAS congeners and predicted probability of having asthma stratified by n-3 PUFA in red cells. Interaction p-value = 0.05 for PFOA and n-3 PUFA and interaction p-value = 0.002 for PFHxS and n-3 PUFA. sex, age, personal income, marital status, smoking status, second-hand smoking, marijuana use, waist circumference, overcrowding, food security, n-3 PUFA quartiles, vitamin D, fruit/vegetable intake, and mercury

Some associations between PFAS and respiratory outcomes were modified by nutritional factors. The association between PFOA and PFHxS in relation to asthma were stronger among individuals in the first and second quartiles of n-3 PUFAs in red blood cells (interaction p-values 0.05 and 0.002, respectively) (Fig. 1 & Supplementary Table 4). The associations between PFDA and PFOS in relation to FEV1/FVC were also stronger in the presence of vitamin D deficiency with steeper slopes (interaction p-values 0.02 and 0.03, respectively) (Supplementary Fig. 2 and Supplementary Table 5).

**Fig. 2:**
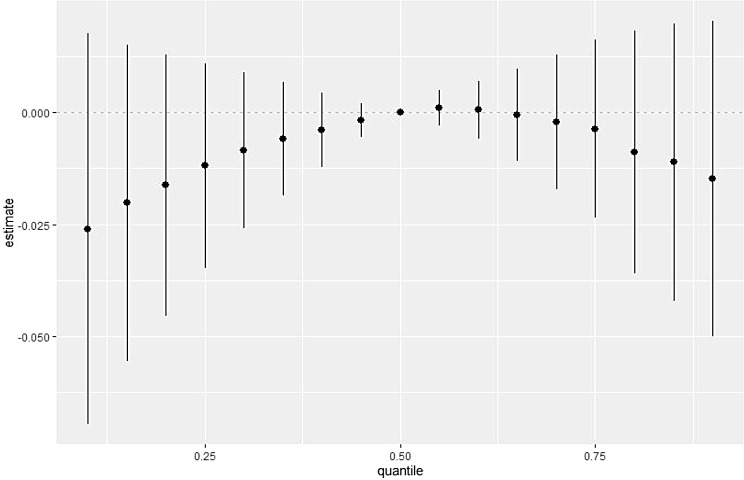
Overall effect of PFAS mixture on asthma based on BKMR. Model is adjusted for sex, age, personal income, marital status, smoking status, second-hand smoking, marijuana use, waist circumference, overcrowding, food security, n-3 PUFA quartiles, vitamin D, fruit/vegetable intake, and mercury

**Fig. 3:**
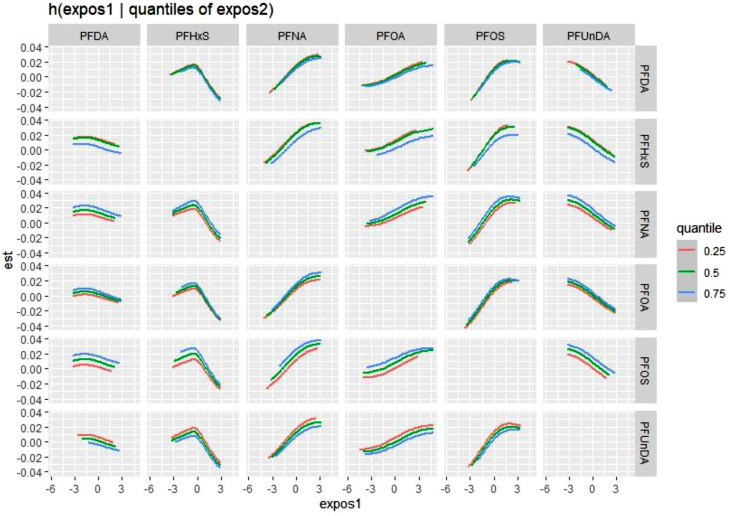
Bivariate exposure-response functions of each PFAS congener and asthma by BKMR analyses. Figures show the associations for each PFAS congener in the column while holding a second PFAS congener in the row constant at different quartiles, and the rest of PFAS congeners at the median. Models are adjusted for sex, age, personal income, marital status, smoking status, second-hand smoking, marijuana use, waist circumference, overcrowding, food security, n-3 PUFA quartiles, vitamin D, fruit/vegetable intake, and mercury concentrations

The overall risk of a PFAS mixture on asthma was not significantly associated with asthma and followed a inverted-U exposure-response curve (Fig. 2). The non-linear association may be driven by the differences in the direction of associations by the type of PFAS congener (Fig. 2). While PFNA, PFOA and PFOS were positively and linearly associated with asthma prevalence (similar to single chemical models), PFUnDA was negatively associated with asthma (Supplementary Fig. 4). PFDA was not associated with asthma, and PFHxS also had a non-linear association with an increase in asthma risk, followed by a decrease in the risk. There was no evidence of interaction between the PFAS congeners (Fig. 3, Supplementary Fig. 3).

Associations were attenuated after excluding participants with emphysema or tuberculosis, but the associations remained positive (Supplementary Table 6). However, the associations were no longer significant after dropping 552 participants with emphysema or tuberculosis in this analysis. No associations were observed between PFAS and asthma when the definition of asthma was expanded to include self-reported asthma, asthmatic bronchitis, allergic bronchitis or COPD (Supplementary Table 7). We still observed associations between PFOS and asthma after restricting the study population to those aged 16–20 years (Supplementary Table 8). Associations between PFAS and FEV1/FVC were attenuated after further adjustment of other persistent organic pollutants (Supplementary Table 9); however, this analysis was restricted to only 500 participants.

## Discussion

We observed an increase in asthma prevalence with higher levels of PFNA and PFOS, as well as a suggestive association with PFOA. PFOA, PFNA, PFDA and PFHxS were associated with a decrease in FEV1/FEV, albeit with low magnitudes of association. No associations were observed between any PFAS congeners and self-reported respiratory symptoms. Some associations were modified by nutritional factors, namely, stronger associations between PFOA and PFHxS and asthma with lower n-3 PUFA levels, and stronger associations between PFDA, PFUnDA and PFOS and FEV1/FVC with vitamin D deficiency. The results add to the growing literature on the potential impact of PFAS on respiratory health, and speak to the importance of country food on Inuit health and continued importance of monitoring PFAS in northern communities.

A study examined the association between PFAS and asthma severity/control (asthma attack and emergency visits) in National Health and Nutrition Examination Survey (NHANES) [14] to account for the inconsistent results from previous studies [53]. There was a general increase in reported asthma attacks in the previous 12 months in adults across all PFAS, but only PFOA reached statistical significance, and associations were stronger among males and those aged < 18 years [14]. We also observed stronger associations between PFOA and asthma compared to other congeners. PFOA concentrations in Nunavik are similar to concentrations in NHANES. No associations were observed between PFAS and emergency care visits for asthma in NHANES.

Although limited evidence is reported linking PFAS and asthma, most studies were conducted in childhood [18, 54] and presented here for the sake of consistency. A cohort in Norway with 675 older adolescents aged 16–18 years (ages included in our current study population) detected strong associations between the sum of PFAS concentrations, PFOS, and PFHxS and asthma (OR 3.35 95% CI 1.54–7.29) [55]. Studies that observed positive associations in children include a case-control Taiwanese study with 456 children aged 10–15 years [56]. Authors reported increased risk of asthma with increased exposure of PFOA, PFDA, PFNA, PFDoA, PFHxS, and PFOS (with an OR 4.05 95% CI 2.21–7.42 for PFOA). An update of this study included additional PFAS congeners and adjusted for further covariates, and still detected a positive association between PFOS, PFOA, PFHxS, PFNA and PFTA [57], albeit with attenuated associations (PFOA: OR 2.76, 95% CI 1.82, 4.17). PFOA was also associated with increased odds of asthma in children aged 12–19 years in NHANES 1999–2000 and 2003–2008, but not PFOS [58]. Moreover, participants aged 3–11 years in NHANES 2013–2014 were more likely to have asthma with concentrations of 2-(N-methyl-perfluorooctane sulfonamido) acetic acid (Me-PFOSA-AcOH), PFDA and PFUnDA above the LOD [17]. Prenatal PFOA, but not PFOS, was associated with self-reported asthma (defined as having at least three wheezing episodes in the 12 months) among five year old children in Denmark, particularly boys [59]. However, no associations were observed with physician-diagnosed asthma [59]. PFHpA was associated with asthma among girls aged 10 years in Oslo; however, this association was not observed when examining the relationship longitudinally at 10 and 16 years [60]. Several other studies found no relationship between PFAS and asthma [61–66].

PFAS were not associated with pulmonary function in adolescent participants of NHANES 2007–2012 [67], in children aged 10 years part of the Taiwan Birth Panel Study [15], nor in children aged 10 and 16 years part of the Environment and Child Asthma Study in Oslo [60]. However, in this later study, PFNA was associated with a decrease in the FEV1/FVC ratio among girls aged 12–15 years, and PFNA was the most heavily weighted chemical after mixture analysis [60]. No associations were observed between childhood exposure to PFAS from living or going to school near the World Trade Center disaster and pulmonary function in adulthood [61], but prenatal exposure to PFOA and PFNA were inversely associated with FEV1 in a study of 1033 children aged 6–12 years part of the European HELIX cohort [68].

We observed evidence of effect modification by n-3 PUFA levels and vitamin D status. Nutritional status has been linked to respiratory health, and although the literature has been mixed, supplements have been studied as potential treatments for various adverse outcomes [69–73]. Several studies showed lower n-3 PUFA levels were associated with increased asthma prevalence, poorer asthma control, and bronchial reactivity [74–79] even though dietary supplementation of n-3 PUFAs may not be associated with better respiratory outcomes [78, 80]. In the present study population, fish and marine mammal consumption (key sources of n-3 PUFA and vitamin D in Nunavik) was associated with increased respiratory symptoms, particularly among those aged 35 years and over [11], which may be explained by the elevated concentrations of environmental contaminants in some Arctic marine species [6, 27]. Consumption of fish and marine mammals is also considered one of the key exposure sources of PFAS [4]. The modifying effects of n-3 PUFAs and vitamin D in our study may point to the protective effect of consuming these foods, despite high PFAS levels in some species and the high PFAS exposure levels in Nunavik compared to other populations [4]. It is important to note that PFOA and PFHxS are the congeners least associated with fish and marine mammal consumption (in comparison to PFDA, PFUnDA and PFOS) [7]. Thus, an interaction effect between congeners more commonly found in these foods (PFDA, PFUnDA, and PFOS) and n-3 PUFAs may have been difficult to detect due to their strong intercorrelations.

Although limited evidence supports the use of vitamin D as a therapeutic measure in preventing asthma exacerbations [81], lower levels of vitamin D are detected among people with asthma or those that experience asthma exacerbations [82]. A meta-analysis on the impact of vitamin D status on lung function in patients with asthma found that patients with low vitamin D levels had lower FEV1 and FEV1/FVC compared to those with sufficient vitamin D levels in both adults and children [83]. Similarly, another review found that most studies detected a positive association between vitamin D levels and lung function [84]. Vitamin D receptors are distributed in respiratory epithelial and immune cells, and may have an immunomodulatory function in respiratory health [83] or influence structural changes in peripheral airways [85].

Mechanisms linking PFAS and adverse lung function are still unclear. Studies have demonstrated impacts on T- and B-cell proliferation, cytokine and antibody production, and decreased respiratory bursts (an indicator of neutrophil function and immunosuppression) [22, 86]. Because of PFAS’ surfactant properties, PFAS could hypothetically alter cell-membrane permeability [19, 87]. An in vitro [87] and a mouse study [19] detected alterations in the membrane properties of BEAS2B cells by PFOA. These authors also showed that the increased mRNA levels of pro-inflammatory and pro-allergic cytokines that short- and long-chain PFAS can induce could led to lung inflammation and subsequent adverse respiratory outcomes [19]. Additionally, in vitro PFOS exposure decreased secretion of T_H_-1 type cytokines (IL-2 and IFN-γ, specifically) and increased T_H_-2 type cytokines (IL-4 and IL-10, specifically), indicating a hallmark of atopy disease with polarization towards T_H_-2 responses, which may contribute to asthma development [88, 89]. Further analyses should examine the mediating effect of immune markers on the association between PFAS and respiratory outcomes.

Another theory points to a potential effect of PFAS on CC16, a small protein secreted by Clara cells. Although the biological function of CC16 remains unclear, the protein can be used as a marker of lung epithelial injury [90]. Pulmonary irritants may cause CC16 levels to decrease, as was shown in studies related to tobacco smoke and arsenic [91, 92]. The decrease in CC16 levels may be due to a reduced number or integrity of Clara cells from PFAS exposure [93]. A detailed description of these pathways was described in Zhou et al. (2017). The same study also found that the association between PFAS and CC16 levels were stronger among asthmatics, pointing to a potential amplification of the effect of PFAS on CC16 levels in the presence of asthma.

Our study makes use of the largest survey of its kind conducted in any Arctic population. The large and representative dataset al.lowed us to examine multiple respiratory outcomes and study the influence of nutritional factors on the relationship between PFAS and adverse respiratory outcomes. However, the cross-sectional design reduces our ability to elucidate causality, particularly regarding asthma. Additionally, despite the high prevalence of some respiratory symptoms (including wheezing), the prevalence of asthma in our survey was low, and may indicate an under-diagnosis of asthma in the region [11]. This, and the high prevalence of smoking, may have reduced our power to detect more associations. Additional studies are required to confirm our results.

## Conclusion

Our study examined the relationship between individual PFAS and a PFAS mixture on respiratory health using data from a large survey based in Nunavik, Quebec. We observed a strong association between PFOA, PFNA and PFOS with diagnosed asthma, but no associations between the overall PFAS mixture and asthma. PFAS were not associated with respiratory symptoms or airway obstruction. Associations were modified by nutritional factors pointing to the nutritional value of traditional Inuit foods. Increased n-3 PUFA and vitamin D levels, two key nutrients found in Nunavik country foods, may attenuate the effect of PFAS on respiratory health in Nunavimmiut. These findings add to the growing literature on the impacts of PFAS on respiratory health, and the importance of their global regulation.

## Electronic supplementary material

Below is the link to the electronic supplementary material.

Supplementary Material 1

## Data Availability

The data are owned by our Inuit colleagues and we do not have permission to share the data.
