# Supplementary material for: Per and poly-fluoroalkyl substances and respiratory health in an Inuit community
Source: Environ Health. 2024 Oct 12;23:83. doi: 10.1186/s12940-024-01126-7 (PMC11470554; doi:10.1186/s12940-024-01126-7)
Supplement: Supplementary file 1 — Supplementary Material 1 [file 12940_2024_1126_MOESM1_ESM.docx]

**Supplementary Material for “Per and poly-fluoroalkyl substances and respiratory health in an Inuit community”**

Amira Aker^1^, Yohann Courtemanche^2^, Pierre Ayotte^2-4^, Philippe Robert^3^, Éric Gaudreau^4^, Mélanie Lemire^2,3,5^

1 School of Public Health, Boston University, Boston, US

2 Axe santé des populations et pratiques optimales en santé, Centre de recherche du CHU de Québec-Université Laval, Québec, Quebec, Canada

3 Département de médecine sociale et préventive, Université Laval, Québec, Quebec, Canada

4 Centre de Toxicologie du Québec, Institut National de Santé Publique du Québec, Québec, Canada

5 Institut de biologie intégrative et des systèmes (IBIS), Université Laval, Quebec, Quebec, Canada

Corresponding author: Amira Aker, Boston University School of Public Health, Department of Epidemiology, 715 Albany Street, Boston, MA 02118; Email: amaker@bu.edu

**Table of Contents**

[Supplementary Table 1: Study population descriptives in the unimputed dataset and imputed and weighted dataset from Q2017, Nunavik, Quebec (N=1326) 4](#_Toc177394864)

[Supplementary Table 2: Odds ratios and their respective 95% confidence intervals of chronic sputum, breathlessness, and any respiratory symptom for every doubling of individual PFAA congener from the Q2017 survey in Nunavik, Quebec. 7](#_Toc177394865)

[Supplementary Table 3: Odds ratios and their respective 95% confidence intervals of respiratory outcomes for every doubling of individual PFAA congener from the Q2017 survey in Nunavik, Quebec, adjusting for sex, age, household income, marital status, smoking status, second-hand smoking, marijuana use, waist circumference, overcrowding, food security, and mercury concentrations (not adjusting for nutritional variables). 8](#_Toc177394866)

[Supplementary Table 4: Interaction p-values between PFAA congener and n-3 PUFA in red blood cells for each outcome. 9](#_Toc177394867)

[Supplementary Table 5: Interaction p-values between PFAA congener and vitamin D deficiency for each outcome 10](#_Toc177394868)

[Supplementary Table 6: Sensitivity analysis removing individuals with tuberculosis (N=447) or emphysema (N=5). Odds ratios or relative risks and their respective 95% confidence intervals of each outcome for every doubling of individual PFAA congener from the Q2017 survey in Nunavik, Quebec. 11](#_Toc177394869)

[Supplementary Table 7: Sensitivity analysis expanding on asthma definition. Odds ratios or relative risks and their respective 95% confidence intervals of each outcome for every doubling of individual PFAA congener from the Q2017 survey in Nunavik, Quebec. 12](#_Toc177394870)

[Supplementary Table 8: Sensitivity analysis restricting analysis by age groups 16-20 years (N=231). Odds ratios or relative risks and their respective 95% confidence intervals of each outcome for every doubling of individual PFAA congener from the Q2017 survey in Nunavik, Quebec. 13](#_Toc177394871)

[Supplementary Table 9: Sensitivity analysis adjusting for other persistent organic pollutants. Odds ratios or relative risks and their respective 95% confidence intervals of each outcome for every doubling of individual PFAA congener from the Q2017 survey in Nunavik, Quebec (N=500). 14](#_Toc177394872)

[Supplementary Figure 1: Directed acyclic graph outlining covariates considered for analysis of PFAAs and respiratory outcomes 6](#_Toc177394873)

[Supplementary Figure 2: Association between PFAA congeners and FEV1/FVC stratified by vitamin D deficiency 17](#_Toc177394874)

[Supplementary Figure 3: The effect of individual PFAAs comparing the changes in each exposure when changing from the 25^th^, 50^th^, and 75^th^ percentiles with the remaining exposures fixed at the 75^th^ percentile 18](#_Toc177394875)

[Supplementary Figure 4: Univariate exposure-response functions for each log2 transformed exposure and asthma, while holding all other exposures at the median value using BKMR 19](#_Toc177394876)

Supplementary Table 1: Study population descriptives in the unimputed dataset and imputed and weighted dataset from Q2017, Nunavik, Quebec (N=1326)

| **Characteristic** | **Not imputed** | **Imputed and weighted** |
| --- | --- | --- |
| **Age (years (SD))** | 37.5 (16.4) | 36.6 (0.19) |
| **Sex** |  |  |
| Female | 65.8 | 49.6 |
| Male | 34.2 | 50.4 |
| **Income** |  |  |
| <$20,000 | 45.5 | 53.7 |
| $20,000-$59,999 | 27.1 | 32.4 |
| >$60,000 | 13.3 | 14.0 |
| Missing | 14.2 | - |
| **Education** |  |  |
| <Grade 9 | 37.0 | 38.8 |
| At least some high school | 45.9 | 47.6 |
| At least some college | 14.1 | 13.6 |
| Missing | 2.9 | - |
| **Marital status** |  |  |
| Married or living with partner | 49.0 | 47.5 |
| Single | 50.8 | 52.5 |
| Missing | 0.2 | - |
| **Smoking** |  |  |
| Never | 10.3 | 10.0 |
| Former | 11.2 | 10.3 |
| Current <15 pack-years | 55.8 | 55.5 |
| Current ≥15 pack-years | 21.0 | 24.3 |
| Missing | 1.6 | - |
| **Second hand smoke** |  |  |
| <1/month | 65.5 | 65.5 |
| 1/week – 1/month | 6.9 | 7.3 |
| Nearly everyday | 26.0 | 27.1 |
| Missing | 1.5 | - |
| **Marijuana use** |  |  |
| Rarely/Never | 23.5 | 26.9 |
| 1-3/month | 17.8 | 20.3 |
| ≥1/week | 41.3 | 52.8 |
| Missing | 17.4 | - |
| **Food security** |  |  |
| Food secure | 32.0 | 34.2 |
| Moderately insecure | 43.9 | 47.0 |
| Severely insecure | 17.0 | 18.8 |
| Missing | 7.1 | - |
| **Housing crowding)** |  |  |
| ≤1 person-per-room | 61.4 | 66.6 |
| > 1 person-per-room | 30.2 | 33.4 |
| Missing | 8.5 | - |
| **n-3 PUFA in red blood cells (%)** |  |  |
| Q1 (<5.83) | 24.9 | 29.2 |
| Q2 (5.83-7.40) | 24.9 | 25.5 |
| Q3 (7.40-9.39) | 24.9 | 23.7 |
| Q4 (>9.39) | 25.0 | 21.6 |
| Missing | 0.4 | - |
| **Fruit/vegetable consumption** |  |  |
| <5 times/day | 74.8 | 83.5 |
| ≥5 times/day | 14.5 | 16.5 |
| Missing | 10.7 | - |
| **Waist Circumference^a^** |  |  |
| Q1 | 23.7 | 24.6 |
| Q2 | 24.3 | 25.1 |
| Q3 | 24.2 | 25.8 |
| Q4 | 24.4 | 24.4 |
| Missing | 3.4 | - |
| ^a^ Waist circumference quartiles based on sex. Females : Q1<82, 82≤Q2<94, 94≤Q3<105, Q4≥105; Males : Q1<79, 79.5≤Q2<86.75, 86.75≤Q3<103.5, Q4≥103.5 cm. | | |


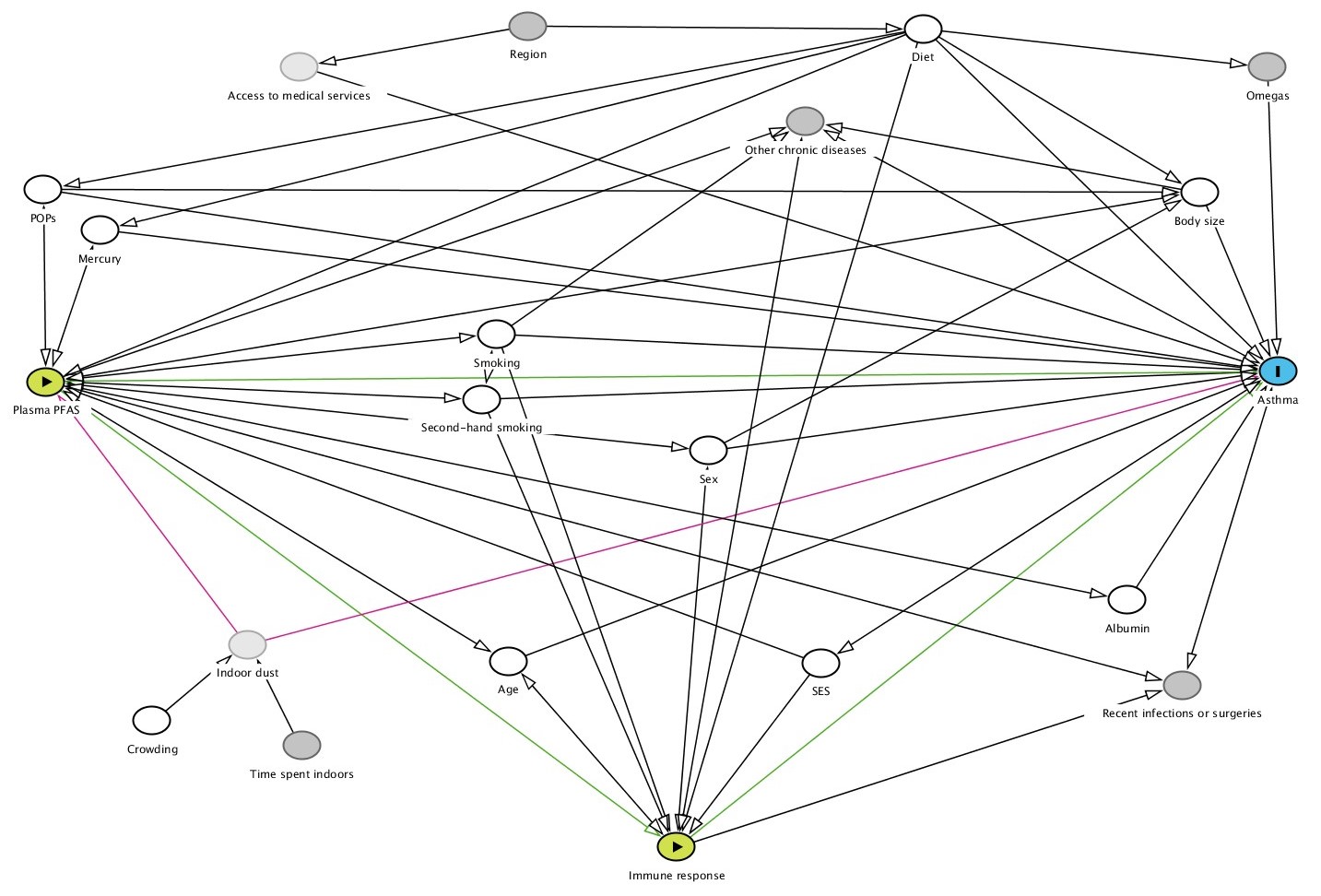


Supplementary Figure 1: Directed acyclic graph outlining covariates considered for analysis of PFAAs and respiratory outcomes

Supplementary Table 2: Odds ratios and their respective 95% confidence intervals of chronic sputum, breathlessness, and any respiratory symptom for every doubling of individual PFAA congener from the Q2017 survey in Nunavik, Quebec.

|  | **Unadjusted** | **Adjusted I** | **Adjusted II** |
| --- | --- | --- | --- |
| **Chronic sputum** |  |  |  |
| PFOA | 1.06 (0.91, 1.25) | 1.02 (0.82, 1.26) | 0.94 (0.75, 1.18) |
| PFNA | 0.98 (0.85, 1.12) | 0.97 (0.83, 1.12) | 0.85 (0.71, 1.01) |
| PFDA | 1.04 (0.94, 1.16) | 1.05 (0.93, 1.19) | 0.88 (0.72, 1.08) |
| PFUnDA | 1.04 (0.93, 1.16) | 1.04 (0.92, 1.18) | 0.85 (0.7, 1.04) |
| PFHxS | 1.07 (0.96, 1.20) | 1.06 (0.91, 1.23) | 0.95 (0.8, 1.14) |
| PFOS | 1.01 (0.91, 1.11) | 1.01 (0.90, 1.14) | 0.87 (0.74, 1.03) |
| **Breathlessness** |  |  |  |
| PFOA | 1.02 (0.87, 1.19) | 1.05 (0.84, 1.30) | 1.07 (0.84, 1.36) |
| PFNA | 0.93 (0.82, 1.07) | 0.91 (0.78, 1.06) | 0.88 (0.73, 1.06) |
| PFDA | 0.99 (0.89, 1.10) | 0.97 (0.85, 1.11) | 0.93 (0.76, 1.13) |
| PFUnDA | 0.98 (0.88, 1.10) | 0.98 (0.86, 1.12) | 0.94 (0.76, 1.15) |
| PFHxS | 1.03 (0.93, 1.16) | 1.06 (0.91, 1.24) | 1.07 (0.89, 1.30) |
| PFOS | 0.97 (0.87, 1.08) | 0.96 (0.84, 1.09) | 0.91 (0.77, 1.09) |
| **Any respiratory symptom** |  |  |  |
| PFOA | 0.99 (0.86, 1.13) | 0.98 (0.81, 1.18) | 0.97 (0.79, 1.18) |
| PFNA | 0.95 (0.85, 1.07) | 0.93 (0.81, 1.06) | 0.88 (0.75, 1.03) |
| PFDA | 1.01 (0.92, 1.11) | 1.00 (0.89, 1.12) | 0.91 (0.76, 1.09) |
| PFUnDA | 1.01 (0.91, 1.11) | 1.00 (0.89, 1.12) | 0.90 (0.75, 1.08) |
| PFHxS | 1.03 (0.93, 1.13) | 1.04 (0.90, 1.20) | 1.01 (0.85, 1.20) |
| PFOS | 0.99 (0.86, 1.13) | 0.98 (0.88, 1.10) | 0.91 (0.78, 1.06) |
| Number of individuals in models = 1298  Adjusted I models are adjusted for sex, age, household income, marital status, smoking status, second-hand smoking, marijuana use, waist circumference, overcrowding, and food security.  Adjusted II models are further adjusted for omega-3 ratio, vitamin D, fruit/vegetable intake, and mercury concentrations. | | | |

Supplementary Table 3: Odds ratios and their respective 95% confidence intervals of respiratory outcomes for every doubling of individual PFAA congener from the Q2017 survey in Nunavik, Quebec, adjusting for sex, age, household income, marital status, smoking status, second-hand smoking, marijuana use, waist circumference, overcrowding, food security, and mercury concentrations (not adjusting for nutritional variables).

|  | **Wheezing** | **Chronic cough** | **Chronic Sputum** |
| --- | --- | --- | --- |
| PFOA | 0.97 (0.78, 1.20) | 1.07 (0.83, 1.37) | 0.96 (0.76, 1.20) |
| PFNA | 0.89 (0.76, 1.04) | 0.96 (0.79, 1.16) | 0.88 (0.74, 1.04) |
| PFDA | 0.97 (0.80, 1.17) | 0.93 (0.75, 1.14) | 0.91 (0.75, 1.10) |
| PFUnDA | 0.92 (0.76, 1.12) | 0.94 (0.76, 1.17) | 0.87 (0.72, 1.06) |
| PFHxS | 0.88 (0.73, 1.05) | 0.98 (0.81, 1.20) | 0.97 (0.81, 1.15) |
| PFOS | 0.92 (0.77, 1.08) | 0.95 (0.79, 1.14) | 0.89 (0.75, 1.05) |
|  | **Breathlessness** | **Asthma** | **Airway obstruction** |
| PFOA | 1.03 (0.82, 1.29) | **1.81 (1.06, 3.10)** | 0.83 (0.62, 1.11) |
| PFNA | 0.86 (0.73, 1.03) | **1.73 (1.23, 2.44)** | 0.85 (0.69, 1.06) |
| PFDA | 0.9 (0.75, 1.09) | 1.50 (1.00, 2.25) | 0.82 (0.64, 1.05) |
| PFUnDA | 0.91 (0.75, 1.12) | 1.38 (0.91, 2.10) | 0.88 (0.68, 1.13) |
| PFHxS | 1.05 (0.88, 1.26) | 1.35 (0.91, 2.02) | 0.83 (0.65, 1.06) |
| PFOS | 0.9 (0.77, 1.07) | **1.53 (1.09, 2.15)** | 0.85 (0.68, 1.06) |
| Number of individuals in models: wheezing, chronic cough, chronic sputum, and breathlessness N=1298; asthma N=1239; airway obstruction N=1110. | | | |

Supplementary Table 4: Interaction p-values between PFAA congener and n-3 PUFA in red blood cells for each outcome.

|  | **Wheezing** | **Chronic cough** | **Chronic Sputum** |
| --- | --- | --- | --- |
| PFOA | 0.96 | 0.69 | 0.63 |
| PFNA | 0.62 | 0.75 | 0.88 |
| PFDA | 0.66 | 0.83 | 0.33 |
| PFUnDA | 0.77 | 0.55 | 0.33 |
| PFHxS | 0.65 | 0.63 | 0.21 |
| PFOS | 0.71 | 0.91 | 0.13 |
|  | **Breathlessness** | **Asthma** | **Airway obstruction** |
| PFOA | 0.30 | **0.05** | 0.43 |
| PFNA | 0.70 | 0.16 | 0.79 |
| PFDA | 0.18 | 0.23 | 0.57 |
| PFUnDA | 0.09 | 0.25 | 0.52 |
| PFHxS | 0.04 | **0.002** | 0.66 |
| PFOS | 0.10 | 0.08 | 0.88 |
|  | **FEV1** | **FVC** | **FEV1/FVC** |
| PFOA | 0.65 | 0.08 | **0.04** |
| PFNA | 0.79 | 0.29 | 0.68 |
| PFDA | 0.98 | 0.35 | 0.82 |
| PFUnDA | 0.80 | 0.66 | 0.82 |
| PFHxS | 0.77 | 0.56 | 0.17 |
| PFOS | 0.92 | 0.15 | 0.53 |
| Interaction term between PFAAs and n-3 PUFA quartiles as an ordinal measure.  Models are adjusted for sex, age, household income, marital status, smoking status, second-hand smoking, marijuana use, waist circumference, overcrowding, food security, omega-3 ratio, vitamin D, fruit/vegetable intake, and mercury concentrations | | | |

Supplementary Table 5: Interaction p-values between PFAA congener and vitamin D deficiency for each outcome

|  | **Wheezing** | **Chronic cough** | **Chronic Sputum** |
| --- | --- | --- | --- |
| PFOA | 0.70 | 0.19 | 0.66 |
| PFNA | 0.93 | 0.88 | 0.50 |
| PFDA | 0.60 | 0.72 | 0.34 |
| PFUnDA | 0.35 | 0.70 | 0.62 |
| PFHxS | 0.64 | 0.16 | 0.74 |
| PFOS | 0.71 | 0.40 | 0.57 |
|  | **Breathlessness** | **Asthma** | **Airway obstruction** |
| PFOA | 0.22 | 0.77 | 0.43 |
| PFNA | 0.22 | 0.68 | 0.79 |
| PFDA | 0.70 | 0.48 | 0.57 |
| PFUnDA | 0.76 | 0.71 | 0.52 |
| PFHxS | 0.14 | 0.57 | 0.66 |
| PFOS | 0.71 | 0.55 | 0.88 |
|  | **FEV1** | **FVC** | **FEV1/FVC** |
| PFOA | 0.99 | 0.93 | 0.57 |
| PFNA | 0.92 | 0.53 | 0.84 |
| PFDA | 0.12 | **0.03** | **0.02** |
| PFUnDA | 0.21 | 0.10 | 0.06 |
| PFHxS | 0.50 | 0.75 | 0.14 |
| PFOS | 0.46 | 0.71 | **0.003** |
| Vitamin D deficiency defined as <30 ng/mL.  Models are adjusted for sex, age, household income, marital status, smoking status, second-hand smoking, marijuana use, waist circumference, overcrowding, food security, omega-3 ratio, vitamin D, fruit/vegetable intake, and mercury concentrations. | | | |

Supplementary Table 6: Sensitivity analysis removing individuals with tuberculosis (N=447) or emphysema (N=5). Odds ratios or relative risks and their respective 95% confidence intervals of each outcome for every doubling of individual PFAA congener from the Q2017 survey in Nunavik, Quebec.

|  | **Wheezing** | **Chronic cough** | **Chronic Sputum** |
| --- | --- | --- | --- |
| PFOA | 0.97 (0.72, 1.31) | 1.39 (1.00, 1.93) | 0.95 (0.72, 1.27) |
| PFNA | 0.92 (0.73, 1.16) | 1.09 (0.84, 1.42) | 0.86 (0.69, 1.08) |
| PFDA | 1.01 (0.76, 1.34) | 0.97 (0.72, 1.31) | 0.87 (0.67, 1.13) |
| PFUnDA | 0.98 (0.74, 1.29) | 1.02 (0.76, 1.37) | 0.85 (0.66, 1.10) |
| PFHxS | 0.87 (0.68, 1.12) | 1.10 (0.83, 1.45) | 0.97 (0.77, 1.23) |
| PFOS | 0.93 (0.73, 1.17) | 0.94 (0.73, 1.20) | 0.84 (0.67, 1.04) |
|  | **Breathlessness** | **Asthma** | **Airway obstruction** |
| PFOA | 1.31 (0.96, 1.79) | 1.71 (0.84, 3.48) | 0.89 (0.60, 1.32) |
| PFNA | 1.06 (0.83, 1.36) | 1.49 (1.00, 2.22) | 0.81 (0.61, 1.07) |
| PFDA | 1.02 (0.78, 1.35) | 1.20 (0.71, 2.05) | 0.80 (0.58, 1.11) |
| PFUnDA | 1.08 (0.81, 1.44) | 1.09 (0.63, 1.87) | 0.87 (0.63, 1.20) |
| PFHxS | 1.27 (0.98, 1.64) | 1.15 (0.70, 1.89) | 0.76 (0.54, 1.07) |
| PFOS | 1.00 (0.79, 1.26) | 1.18 (0.77, 1.82) | 0.84 (0.63, 1.11) |
|  | **FEV1** | **FVC** | **FEV1/FVC** |
| PFOA | 1.05 (1.01, 1.09) | 1.01 (0.99, 1.04) | 0.98 (0.97, 1.00) |
| PFNA | 1.00 (0.98, 1.03) | 0.99 (0.97, 1.01) | 0.99 (0.98, 1.00) |
| PFDA | 0.98 (0.95, 1.02) | 1.00 (0.97, 1.02) | 0.99 (0.98, 1.00) |
| PFUnDA | 0.99 (0.95, 1.02) | 1.00 (0.98, 1.03) | 0.99 (0.98, 1.00) |
| PFHxS | 1.02 (0.99, 1.06) | 1.01 (0.99, 1.03) | 0.99 (0.98, 1.00) |
| PFOS | 1.00 (0.98, 1.03) | 1.01 (0.99, 1.03) | 0.99 (0.98, 1.00) |
| Models are adjusted for sex, age, household income, marital status, smoking status, second-hand smoking, marijuana use, waist circumference, overcrowding, food security, omega-3 ratio, vitamin D, fruit/vegetable intake, and mercury concentrations. | | | |

Supplementary Table 7: Sensitivity analysis expanding on asthma definition. Odds ratios or relative risks and their respective 95% confidence intervals of each outcome for every doubling of individual PFAA congener from the Q2017 survey in Nunavik, Quebec.

|  | **Asthma, Asthmatic Bronchitis, or Allergic Bronchitis** | **Asthma, Asthmatic Bronchitis, Allergic Bronchitis, or COPD** |
| --- | --- | --- |
| PFOA | 0.97 (0.72, 1.31) | 1.39 (1.00, 1.93) |
| PFNA | 0.92 (0.73, 1.16) | 1.09 (0.84, 1.42) |
| PFDA | 1.01 (0.76, 1.34) | 0.97 (0.72, 1.31) |
| PFUnDA | 0.98 (0.74, 1.29) | 1.02 (0.76, 1.37) |
| PFHxS | 0.87 (0.68, 1.12) | 1.10 (0.83, 1.45) |
| PFOS | 0.93 (0.73, 1.17) | 0.94 (0.73, 1.20) |
| Participants with self-reported asthma, asthmatic bronchitis, or allergic bronchitis: N=59. Participants with chronic obstructive pulmonary disease (COPD): N=89.  Models are adjusted for sex, age, household income, marital status, smoking status, second-hand smoking, marijuana use, waist circumference, overcrowding, food security, omega-3 ratio, vitamin D, fruit/vegetable intake, and mercury concentrations. | | |

Supplementary Table 8: Sensitivity analysis restricting analysis by age groups 16-20 years (N=231). Odds ratios or relative risks and their respective 95% confidence intervals of each outcome for every doubling of individual PFAA congener from the Q2017 survey in Nunavik, Quebec.

|  | **16-20 years** |
| --- | --- |
| PFOA | 4.51 (0.91, 22.2) |
| PFNA | 2.56 (0.91, 7.20) |
| PFDA | 3.13 (0.98, 10.02) |
| PFUnDA | 2.91 (0.85, 10.01) |
| PFHxS | 3.67 (0.78, 17.33) |
| PFOS | **3.32 (1.08, 10.24)** |
| Models are adjusted for sex, age, household income, marital status, smoking status, second-hand smoking, marijuana use, waist circumference, overcrowding, food security, omega-3 ratio, vitamin D, fruit/vegetable intake, and mercury concentrations. | |

Supplementary Table 9: Sensitivity analysis adjusting for other persistent organic pollutants. Odds ratios or relative risks and their respective 95% confidence intervals of each outcome for every doubling of individual PFAA congener from the Q2017 survey in Nunavik, Quebec (N=500).

|  |  | **No adjustment** | **Chlordane** | **DDE** | **HCH** | **Hexachloro-benzene** | **Mirex** | **Toxaphene** | **PCBs** |
| --- | --- | --- | --- | --- | --- | --- | --- | --- | --- |
| **Wheezing** | **PFOA** | 1.01  (0.80, 1.26) | 1.17  (0.84, 1.63) | 1.16  (0.83, 1.62) | 1.13  (0.82, 1.57) | 1.16  (0.83, 1.62) | 1.17  (0.84, 1.63) | 1.17  (0.84, 1.63) | 1.15  (0.83, 1.61) |
|  | **PFNA** | 0.91  (0.77, 1.08) | 0.83  (0.63, 1.1) | 0.82  (0.62, 1.09) | 0.81  (0.62, 1.06) | 0.81  (0.61, 1.08) | 0.85  (0.65, 1.12) | 0.84  (0.64, 1.11) | 0.81  (0.61, 1.08) |
|  | **PFDA** | 0.99  (0.85, 1.15) | 0.87  (0.66, 1.14) | 0.86  (0.66, 1.12) | 0.85  (0.66, 1.09) | 0.85  (0.64, 1.12) | 0.90  (0.70, 1.17) | 0.89  (0.68, 1.15) | 0.84  (0.64, 1.10) |
|  | **PFUnDA** | 0.96  (0.83, 1.11) | 0.84  (0.65, 1.09) | 0.84  (0.65, 1.08) | 0.83  (0.65, 1.05) | 0.82  (0.63, 1.07) | 0.88  (0.69, 1.12) | 0.86  (0.67, 1.11) | 0.82  (0.64, 1.06) |
|  | **PFHxS** | 0.90  (0.76, 1.06) | 0.80  (0.59, 1.07) | 0.79  (0.59, 1.05) | 0.77  (0.58, 1.03) | 0.78  (0.58, 1.05) | 0.82  (0.62, 1.09) | 0.81  (0.61, 1.08) | 0.77  (0.57, 1.04) |
|  | **PFOS** | 0.94  (0.82, 1.08) | 0.82  (0.63, 1.07) | 0.81  (0.62, 1.05) | 0.80  (0.63, 1.02) | 0.80  (0.62, 1.05) | 0.85  (0.66, 1.09) | 0.84  (0.65, 1.08) | 0.79  (0.61, 1.04) |
| **Chronic cough** | **PFOA** | 1.13  (0.87, 1.45) | 1.23  (0.83, 1.82) | 1.26  (0.85, 1.87) | 1.23  (0.84, 1.81) | 1.27  (0.86, 1.88) | 1.26  (0.85, 1.85) | 1.24  (0.84, 1.83) | 1.27  (0.86, 1.87) |
|  | **PFNA** | 1.02  (0.85, 1.23) | 0.96  (0.71, 1.31) | 0.99  (0.73, 1.35) | 0.97  (0.71, 1.33) | 1.00  (0.73, 1.35) | 0.99  (0.72, 1.36) | 0.98  (0.72, 1.33) | 0.99  (0.72, 1.36) |
|  | **PFDA** | 1.05  (0.89, 1.24) | 1.05  (0.78, 1.41) | 1.11  (0.83, 1.47) | 1.07  (0.8, 1.42) | 1.12  (0.83, 1.51) | 1.10  (0.80, 1.50) | 1.08  (0.8, 1.44) | 1.12  (0.82, 1.52) |
|  | **PFUnDA** | 1.07  (0.92, 1.26) | 1.13  (0.85, 1.49) | 1.18  (0.89, 1.56) | 1.13  (0.86, 1.49) | 1.20  (0.90, 1.59) | 1.17  (0.87, 1.58) | 1.15  (0.87, 1.51) | 1.19  (0.89, 1.6) |
|  | **PFHxS** | 1.06  (0.88, 1.27) | 0.94  (0.70, 1.28) | 0.98  (0.72, 1.33) | 0.95  (0.71, 1.29) | 0.98  (0.73, 1.33) | 0.98  (0.72, 1.33) | 0.97  (0.73, 1.30) | 0.98  (0.72, 1.34) |
|  | **PFOS** | 1.04  (0.90, 1.20) | 1.00  (0.76, 1.3) | 1.04  (0.79, 1.37) | 1.01  (0.77, 1.31) | 1.05  (0.8, 1.37) | 1.03  (0.77, 1.38) | 1.02  (0.79, 1.32) | 1.05  (0.79, 1.38) |
| **Chronic sputum** | **PFOA** | 0.96  (0.77, 1.20) | 0.98  (0.68, 1.4) | 0.97  (0.67, 1.40) | 0.96  (0.68, 1.38) | 0.96  (0.67, 1.39) | 1.02  (0.72, 1.46) | 0.98  (0.69, 1.40) | 0.99  (0.69, 1.43) |
|  | **PFNA** | 0.89  (0.75, 1.05) | 0.77  (0.56, 1.06) | 0.77  (0.56, 1.06) | 0.77  (0.57, 1.05) | 0.75  (0.55, 1.04) | 0.84  (0.61, 1.15) | 0.79  (0.58, 1.08) | 0.79  (0.57, 1.08) |
|  | **PFDA** | 0.99  (0.85, 1.14) | 0.87  (0.65, 1.17) | 0.86  (0.65, 1.15) | 0.86  (0.65, 1.15) | 0.84  (0.63, 1.13) | 0.99  (0.73, 1.35) | 0.89  (0.67, 1.19) | 0.9  (0.66, 1.22) |
|  | **PFUnDA** | 0.97  (0.84, 1.13) | 0.91  (0.68, 1.23) | 0.90  (0.68, 1.20) | 0.90  (0.69, 1.18) | 0.88  (0.66, 1.18) | 1.03  (0.76, 1.38) | 0.94  (0.70, 1.24) | 0.94  (0.70, 1.26) |
|  | **PFHxS** | 1.00  (0.85, 1.18) | 1.00  (0.75, 1.34) | 0.99  (0.74, 1.33) | 0.99  (0.74, 1.32) | 0.98  (0.73, 1.32) | 1.09  (0.81, 1.46) | 1.01  (0.76, 1.34) | 1.03  (0.76, 1.39) |
|  | **PFOS** | 0.96  (0.84, 1.10) | 0.92  (0.71, 1.19) | 0.91  (0.70, 1.18) | 0.91  (0.70, 1.17) | 0.90  (0.69, 1.16) | 1.03  (0.79, 1.35) | 0.94  (0.73, 1.20) | 0.94  (0.72, 1.23) |
| **Breathlessness** | **PFOA** | 1.07  (0.85, 1.35) | 1.09  (0.72, 1.66) | 1.12  (0.74, 1.69) | 1.12  (0.74, 1.7) | 1.13  (0.74, 1.71) | 1.16  (0.77, 1.76) | 1.10  (0.73, 1.66) | 1.11  (0.73, 1.69) |
|  | **PFNA** | 0.89  (0.75, 1.06) | **0.69**  **(0.48, 0.99)** | 0.70  (0.49, 1.00) | 0.73  (0.51, 1.03) | 0.71  (0.5, 1.02) | 0.75  (0.53, 1.06) | 0.71  (0.5, 1.00) | **0.69**  **(0.49, 0.99)** |
|  | **PFDA** | 0.96  (0.81, 1.12) | 0.97  (0.68, 1.37) | 1.01  (0.73, 1.41) | 1.02  (0.74, 1.41) | 1.03  (0.73, 1.46) | 1.10  (0.77, 1.56) | 0.97  (0.70, 1.35) | 1.00  (0.70, 1.42) |
|  | **PFUnDA** | 0.97  (0.83, 1.13) | 0.97  (0.7, 1.34) | 1.02  (0.75, 1.39) | 1.02  (0.75, 1.38) | 1.03  (0.75, 1.42) | 1.10  (0.79, 1.52) | 0.97  (0.71, 1.32) | 1.00  (0.72, 1.4) |
|  | **PFHxS** | 1.06  (0.89, 1.27) | 1.06  (0.76, 1.48) | 1.09  (0.79, 1.52) | 1.09  (0.79, 1.52) | 1.11  (0.79, 1.55) | 1.16  (0.82, 1.65) | 1.07  (0.77, 1.47) | 1.08  (0.76, 1.54) |
|  | **PFOS** | 0.94  (0.81, 1.09) | 0.87  (0.63, 1.18) | 0.90  (0.66, 1.22) | 0.92  (0.69, 1.23) | 0.92  (0.68, 1.25) | 0.98  (0.72, 1.33) | 0.88  (0.66, 1.18) | 0.89  (0.65, 1.22) |
| **Airway Obstruction** | **PFOA** | 0.86  (0.64, 1.16) | 0.90  (0.52, 1.53) | 0.88  (0.51, 1.49) | 0.89  (0.53, 1.51) | 0.93  (0.54, 1.58) | 0.84  (0.5, 1.4) | 0.88  (0.52, 1.5) | 0.88  (0.52, 1.5) |
|  | **PFNA** | 0.92  (0.74, 1.14) | 0.86  (0.58, 1.28) | 0.84  (0.57, 1.24) | 0.86  (0.58, 1.27) | 0.90  (0.6, 1.33) | 0.80  (0.55, 1.17) | 0.85  (0.58, 1.25) | 0.85  (0.57, 1.26) |
|  | **PFDA** | 0.99  (0.82, 1.2) | 1.10  (0.75, 1.61) | 1.03  (0.72, 1.46) | 1.05  (0.74, 1.50) | 1.16  (0.8, 1.7) | 0.95  (0.68, 1.33) | 1.05  (0.73, 1.52) | 1.05  (0.73, 1.52) |
|  | **PFUnDA** | 1.05  (0.87, 1.26) | 1.20  (0.84, 1.74) | 1.11  (0.79, 1.55) | 1.13  (0.81, 1.59) | 1.27  (0.88, 1.83) | 1.03  (0.75, 1.41) | 1.16  (0.81, 1.65) | 1.14  (0.81, 1.6) |
|  | **PFHxS** | 0.92  (0.73, 1.17) | 1.03  (0.65, 1.61) | 0.99  (0.64, 1.54) | 1.02  (0.66, 1.58) | 1.08  (0.68, 1.71) | 0.94  (0.61, 1.43) | 1.00  (0.65, 1.53) | 1.01  (0.64, 1.59) |
|  | **PFOS** | 0.98  (0.82, 1.17) | 0.97  (0.68, 1.36) | 0.93  (0.67, 1.3) | 0.96  (0.69, 1.33) | 1.01  (0.71, 1.43) | 0.86  (0.61, 1.22) | 0.94  (0.68, 1.3) | 0.94  (0.67, 1.33) |
| **FEV1/FVC** | **PFOA** | **-0.02**  **(-0.03, 0)** | **-0.02**  **(-0.05, 0)** | **-0.02**  **(-0.04, 0)** | **-0.02**  **(-0.04, 0)** | **-0.02**  **(-0.04, 0)** | **-0.03**  **(-0.05, -0.01)** | **-0.02**  **(-0.05, 0)** | **-0.02**  **(-0.05, 0)** |
|  | **PFNA** | **-0.01**  **(-0.02, 0)** | -0.01  (-0.03, 0.01) | -0.01  (-0.03, 0.01) | -0.01  (-0.02, 0.01) | -0.01  (-0.02, 0.01) | **-0.02**  **(-0.03, 0)** | -0.01  (-0.03, 0.01) | -0.01  (-0.03, 0.01) |
|  | **PFDA** | **-0.01**  **(-0.02, 0)** | 0.005  (-0.01, 0.02) | 0.004  (-0.01, 0.02) | 0.01  (-0.01, 0.02) | 0.01  (-0.01, 0.03) | -0.01  (-0.02, 0.01) | 0.003  (-0.01, 0.02) | 0.001  (-0.02, 0.02) |
|  | **PFUnDA** | **-0.01**  **(-0.02, 0)** | 0.01  (-0.01, 0.02) | 0.005  (-0.01, 0.02) | 0.01  (-0.01, 0.02) | 0.01  (-0.01, 0.03) | -0.01  (-0.02, 0.01) | 0.004  (-0.01, 0.02) | 0.001  (-0.02, 0.02) |
|  | **PFHxS** | **-0.01**  **(-0.02, 0)** | -0.01  (-0.03, 0.01) | -0.01  (-0.03, 0.01) | -0.01  (-0.02, 0.01) | -0.01  (-0.02, 0.01) | -0.02  (-0.03, 0) | -0.01  (-0.03, 0.01) | -0.01  (-0.03, 0.01) |
|  | **PFOS** | **-0.01  (-0.01, 0.00)** | 0.004  (-0.01, 0.02) | 0.002  (-0.01, 0.02) | 0.01  (-0.01, 0.02) | -0.01  (-0.02, 0.01) | -0.005  (-0.01, 0.02) | 0.002  (-0.01, 0.02) | 0.001 (-0.02, 0.02) |
| Models are adjusted for sex, age, household income, marital status, smoking status, second-hand smoking, marijuana use, waist circumference, overcrowding, food security, omega-3 ratio, vitamin D and fruit/vegetable intake.  Bolded associations represent associations with p-values<0.05. | | | | | | | | | |


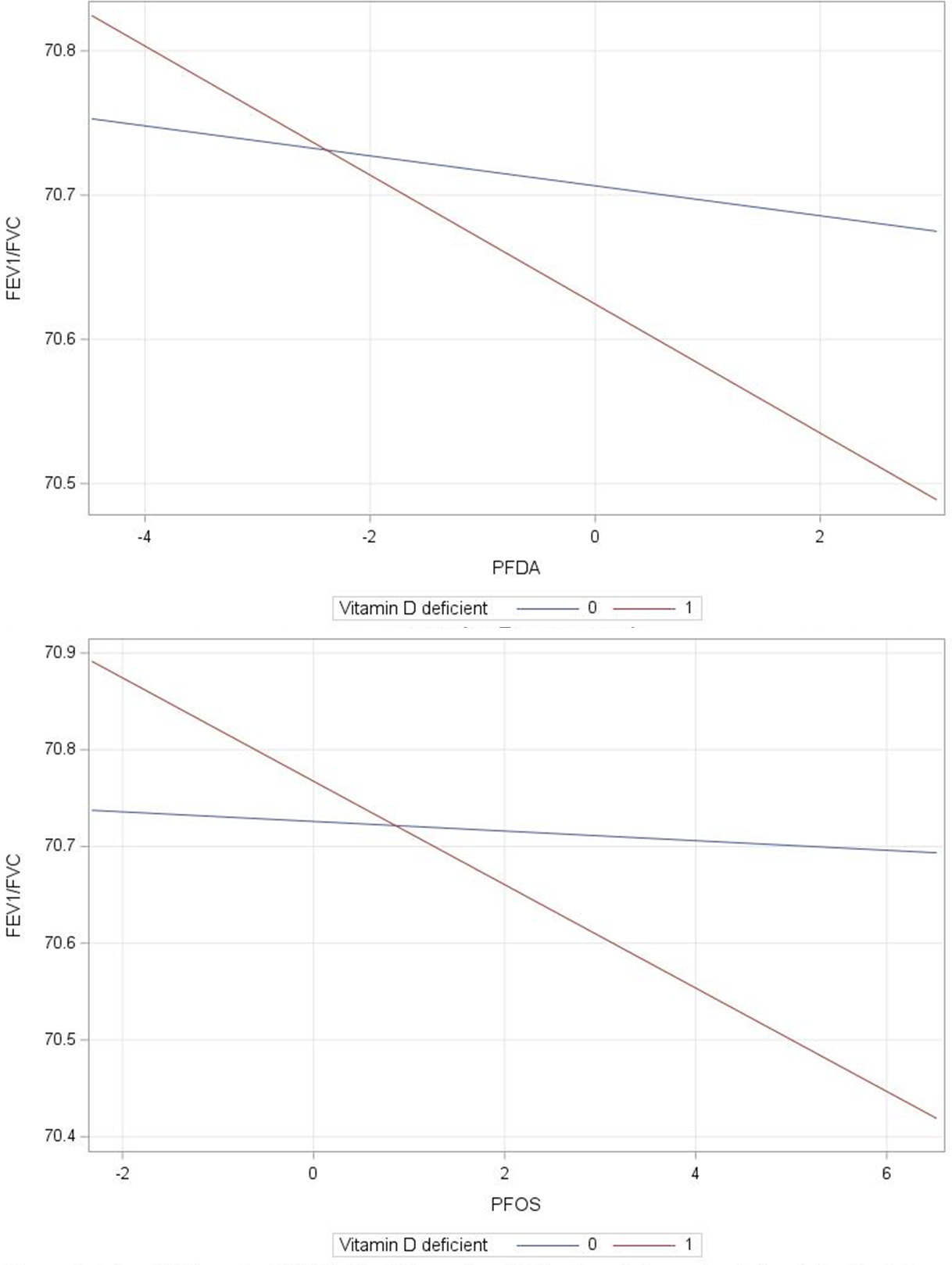


Supplementary Figure 2: Association between PFAA congeners and FEV1/FVC stratified by vitamin D deficiency. Interaction p-value = 0.02 for PFDA and vitamin D deficiency and interaction p-value = 0.003 for PFOS and vitamin D deficiency


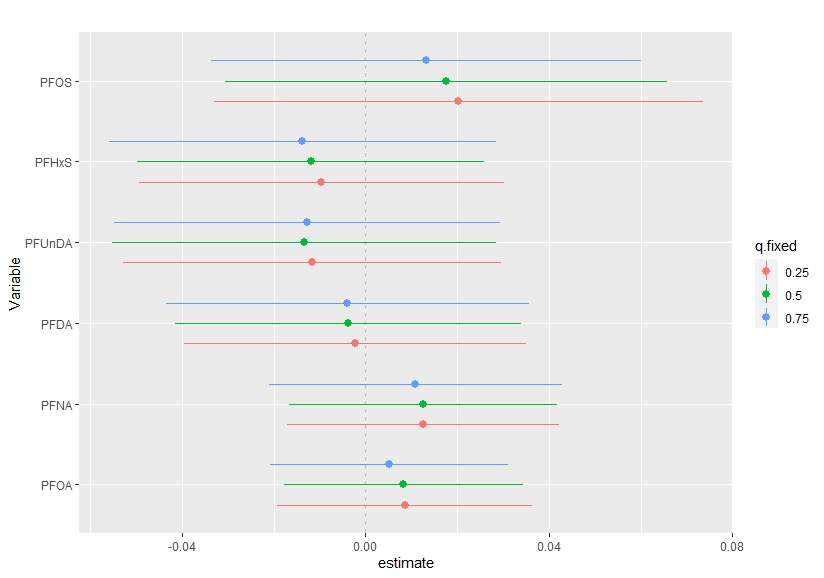


Supplementary Figure 3: The effect of individual PFAAs comparing the changes in each exposure when changing from the 25^th^, 50^th^, and 75^th^ percentiles with the remaining exposures fixed at the 75^th^ percentile. Models adjusted for sex, age, household income, marital status, smoking status, second-hand smoking, marijuana use, waist circumference, overcrowding, food security, omega-3 ratio, vitamin D, fruit/vegetable intake, and mercury concentrations.


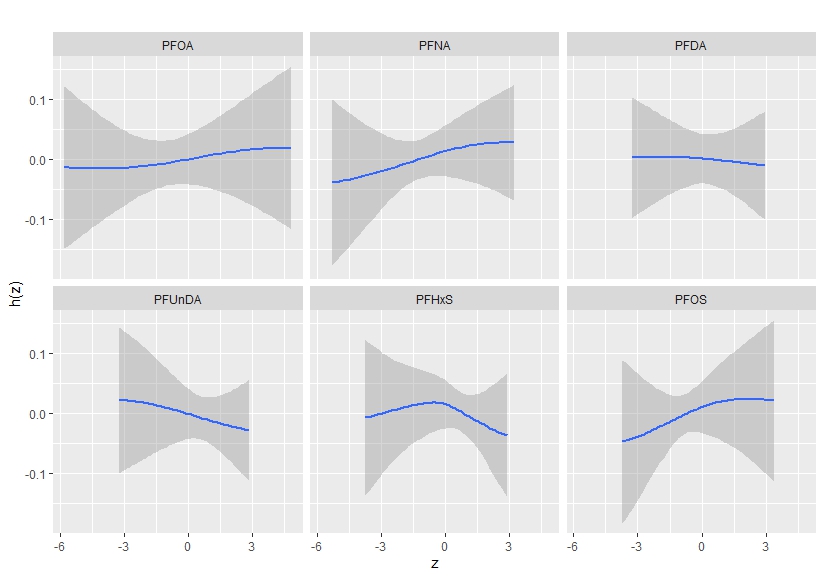


Supplementary Figure 4: Univariate exposure-response functions for each log2 transformed exposure and asthma, while holding all other exposures at the median value using BKMR. Models adjusted for sex, age, household income, marital status, smoking status, second-hand smoking, marijuana use, waist circumference, overcrowding, food security, omega-3 ratio, vitamin D, fruit/vegetable intake, and mercury concentrations.
